# Rapamycin improves the quality and developmental competence of mice oocytes by promoting DNA damage repair during in vitro maturation

**DOI:** 10.1186/s12958-022-00943-0

**Published:** 2022-04-18

**Authors:** Qiyu Yang, Qingsong Xi, Meng Wang, Rui Long, Juan Hu, Zhou Li, Xinling Ren, Lixia Zhu, Lei Jin

**Affiliations:** Reproductive Medicine Center, Tongji Hospital, Tongji Medical College, Huazhong University of Science and Technology, No.1095, Jiefang Road, Wuhan, 430030 China

**Keywords:** In vitro maturation, Rapamycin, mTOR, Oocyte quality, DNA damage

## Abstract

**Background:**

Increasing evidence has shown that the mammalian target of rapamycin (mTOR) pathway plays a critical role in oocyte meiosis and embryonic development, however, previous studies reporting the effects of rapamycin on oocyte IVM showed different or even opposite results, and the specific mechanisms were not clear.

**Methods:**

The immature oocytes from female mice underwent IVM with rapamycin at different concentrations to select an optimal dose. The maturation rate, activation rate, subsequent cleavage and blastocyst formation rates, spindle assembly, chromosome alignment, mitochondrial membrane potential (MMP), ROS levels, and DNA damage levels were evaluated and compared in oocytes matured with or without rapamycin. In addition, the expression levels of genes associated with mTORC1 pathway, spindle assembly, antioxidant function, and DNA damage repair (DDR) were also assessed and compared.

**Results:**

Rapamycin at 10 nM was selected as an optimal concentration based on the higher maturation and activation rate of IVM oocytes. Following subsequent culture, cleavage and blastocyst formation rates were elevated in activated embryos from the rapamycin group. Additionally, oocytes cultured with 10 nM rapamycin presented decreased ROS levels, reduced chromosome aberration, and attenuated levels of γ-H2AX. No significant effects on the percentages of abnormal spindle were observed. Correspondingly, the expressions of *Nrf2*, *Atm*, *Atr*, and *Prkdc* in IVM oocytes were markedly increased, following the inhibition of mTORC1 pathway by 10 nM rapamycin.

**Conclusion:**

Rapamycin at 10 nM could ameliorate the developmental competence and quality of IVM oocytes of mice, mainly by improving the chromosome alignments. The inhibition of mTORC1 pathway, which involved in activating DDR-associated genes may act as a potential mechanism for oocyte quality improvement by rapamycin.

**Supplementary Information:**

The online version contains supplementary material available at 10.1186/s12958-022-00943-0.

## Introduction

Oocyte in vitro maturation (IVM) is a rapidly developing technique of reproductive medicine in the past three decades. It is not only a supplement to in vitro fertilization-embryo transfer (IVF-ET) technique to increase the available embryo rate, but also an effective method to avoid ovarian hyperstimulation syndrome (OHSS) in patients with polycystic ovary syndrome (PCOS), by reducing the hormone dosage during controlled ovarian hyperstimulation (COH). Besides, IVM is one of the fertility preservation methods for cancer patients, which could eliminate the risk of re-implantation of tumor cells during ovarian tissue cryopreservation and transplantation [1, 2]. Since human immature oocytes were initially cultured by IVM technology, resulting in a live birth after fertilization in 1991 [3], many studies regarding the establishment of IVM system have been reported, and some reproductive centers are also applying this technology in clinical practice, achieving different success rates [4].

However, it is a consensus that embryos derived from IVM oocytes have lower developmental competence than sibling embryos derived from oocytes matured in vivo, which may be attributed to the metabolic disorder, oxidative stress, and increased DNA double-strand breaks (DSBs) or chromosome segregation suffered by IVM oocytes [5]. Minimizing the damage to oocytes caused by the in vitro environment is also an important method of improving IVM outcomes.

Rapamycin is a macrolide metabolite produced by *Streptomyces hygroscopicus*, which was first identified in 1975, with antifungal and immunosuppressive effects [6, 7]. The mammalian target of rapamycin (mTOR) is sensitive to various environmental factors such as nutrients and growth factors, and regulates important processes such as cell growth, metabolism, differentiation, aging, and autophagy [8]. In recent years, increasing evidence has shown that the mTOR pathway also plays a critical role in important female reproductive activities, including folliculogenesis [9], oocyte meiosis [10], ovarian aging [11], and embryonic development [12].

Previous studies reported that the addition of rapamycin affected the outcomes of oocyte IVM, but showed opposite effects at different concentrations in various species. Several studies demonstrated negative impacts of rapamycin at concentrations of 0.5-10 μg/μl (i.e. 0.55-10.94 mM) or 10 μM on IVM oocytes [13, 14], while others reported that 1 nM or 100 nM rapamycin in IVM could improve the nuclear maturation rate, cytoplasmic maturation, and subsequent developmental potential of oocytes [15–19]. Based on these results, we speculated that the effect of rapamycin on IVM oocytes might be dose-dependent.

Therefore, one purpose of the current study is to design a large-range concentration gradient screening to clarify the optimal concentration, at which rapamycin exerts its positive effect. Moreover, we also aim to specifically estimate the effects of rapamycin on the quality and developmental competence of IVM oocytes and to preliminarily explore the potential mechanisms.

## Materials and methods

### Ethics approval

All the procedures for animal care and handling were conducted in accordance with the guidelines and approved by the Ethical Committee of Tongji Hospital, Tongji Medicine College, Huazhong University of Science and Technology (TJ-IRB20201209).

### Oocytes collection and in vitro maturation

ICR mice were purchased from Beijing SiPeiFu Biotechnology Co., Ltd. and raised in a room with 14 h light: 10 h dark cycles. Female mice, at the age of 8–10 weeks, were sacrificed by cervical dislocation. The bilateral ovaries were taken and transferred to the M2 medium (EasyCheck, M1250) containing 50 μM isobutylmethylxanthine (IBMX) (Selleck, S5836), and oocytes were retrieved by puncturing the ovaries with sterile needles. The cumulus cells surrounding oocytes were removed by repeated mechanical pipetting. After being washed thoroughly in M2 droplets, the denuded GV oocytes were transferred to 20 μl droplets of IVM medium (EasyCheck, M2115) and cultured at 37 °C under 5% CO_2_ in humidified air. Sixteen hours later, MII oocytes were identified with the extrusion of PB1, and maturation rates of each group were recorded.

During the experiments with only rapamycin (MedChemExpress, HY-10219, 10 mM in 1 ml DMSO), it is diluted with M2 medium, so the final concentrations of DMSO ranged from 1‰ to 0.0001‰, which were negligible. During the experiment adding 250 μM MHY1485 (mTOR activator, MedChemExpress, HY-B0795), the final concentration of DMSO in MHY1485 group was 2.5%, so the same concentration of DMSO was added to both the control and rapamycin groups to avoid impact from confounding factor.

### Parthenogenetic activation (PA) of in vitro matured oocytes

The matured oocytes were first treated with 5 μM ionomycin in M2 medium for 7 min, and then cultured for 4 h in CZB medium (EasyCheck, M1650) supplemented with 2 mM 6-DMAP (Sigma, D2629) and 5 mg/l cytochalasin B (MedChemExpress, HY-16928), at 37 °C in a humidified atmosphere containing 5% CO_2_ in air. Following activation, the oocytes were washed and transferred to CZB droplets for subsequent culture. After 24 h, oocytes were observed under an inverted microscope for activation assessment. Oocytes showing two symmetrical cells each having a nucleolus were judged as activated. Activated embryos were further cultured in CZB and the cleavage rate and blastocyst formation rate were assessed at 48 h and 108 h, respectively.

### Immunofluorescence analysis

For spindle and chromosome staining, the in vitro matured oocytes were 1) fixed in 4% paraformaldehyde (Servicebio, G1101) for 30 min at 37 °C; 2) permeabilized with 0.1% Triton X-100 (Solarbio, T8200) for 10 min; 3) washed three times in 1% bovine serum albumin (BSA) and blocked for 1 h in 3% BSA; 4) incubated overnight at 4 °C with primary antibodies (1:100 diluted anti-mouse β-tubulin antibody, CST, 86298S; 1:200 diluted anti-rabbit phospho-histone H2AX antibody, CST, 9718S); 5) washed three times in 1% BSA and incubated in goat anti-mouse IgG labeled with fluorescein isothiocyanate (1:200, Proteintech, SA00003-1) and goat anti-rabbit IgG labeled with cyanine 3 (1:200, Servicebio, GB21303). Then, the oocytes were incubated for 10 min with 4 μg/ml Hoechst 33258 (Servicebio, G1011). The stained oocytes were mounted onto glass slides and examined under a fluorescence microscope (Axio Observer A1; Carl Zeiss, Germany).

The normal spindle is spindle-shaped and symmetric with a prominent pole on each side, while the abnormal spindle is disintegrated, with a shortened length. The normal chromosome is aggregated and aligned orderly on the equatorial plate, while the abnormal chromosomes might separate from the equatorial plate, being excessively loose or scattered distribution.

For measurement of intra-oocyte reactive oxygen species (ROS), the stock solution of 2′,7′- dichlorodihydro- fluorescein diacetate (DCHF-DA) (Sigma, D6883) was diluted to 10 μM with M2 medium, and MII oocytes were stained with the resultant solution at 37 °C for 20 min. After washing three times in M2 to remove excess dye, the oocytes were immediately examined under a microscope with fluorescence obtained by excitation at 488 nm.

For measurement of mitochondrial membrane potential (MMP), a MMP detection (JC-1) kit (Beyotime Biotechnology Research Institute, China) was applied. Briefly, MII oocytes were washed three times in the M2 medium and placed in a drop of 50 μl M2 and 50 μl JC-1 dye working solution. After being incubated at 37 °C for 25 min, the oocytes were washed three times in JC-1 staining buffer and immediately observed under a fluorescence microscope. The same oocytes were observed through the TRITC channel (red fluorescence) and FITC channel (green fluorescence). The ratio of aggregated JC-1 (red fluorescence) to monomeric JC-1 (green fluorescence) was calculated to quantify changes in MMP, with a decreased red/green JC-1 ratio representing depolarization of the mitochondria.

For measurement of immunofluorescence intensity of γH2AX, ROS, and mitochondria, all the images of different groups were taken using fixed microscopic parameters and the experiment was repeated three independent times with each group involving 20 to 25 oocytes. The images were analyzed with Image J software (NIH, Bethesda, MD, USA).

### Real-time quantitative PCR

The extraction of total mRNA from MII oocytes was conducted using NucleoZol reagent (Macherey-Nagel, Germany). Reverse transcription of the extracted mRNA was performed using HiScript II Q RT SuperMix for qPCR (+gDNA wiper) (Vazyme, R223-01). The PCR reaction mixture contained 10 μl ChamQ Universal SYBR qPCR Master Mix (Vazyme, Q711-02/03), 1 μl PCR Forward Primer (10 μM), 1 μl PCR Reverse Primer (10 μM), 1 μl prepared cDNA, and 7 μl RNase-free water. The primer pairs for real-time PCR are listed in Supplemental Table 1. PCR specificity was identified by analyzing melting curve data and gene expressions were normalized by comparison to the GAPDH transcriptional level.

### Statistical analyses

Data from at least three replicates were expressed as mean ± SEM and analyzed by means of the analysis of variance with the use of the GraphPad Prism 8.0 (San Diego, CA, USA), followed by a Chi-square test. *P* < 0.05 was considered to be statistically significant.

## Results

### The optimal concentration of rapamycin in IVM

To determine the optimal concentration of rapamycin, GV oocytes of mice were cultured in IVM medium supplemented with different concentrations (0 nM, 1 nM, 10 nM, 100 nM, 1 μM, 10 μM) of rapamycin for 16 h, and then activated respectively. As shown in Table 1, the maturation rate and activation rate of oocytes cultured with 10 nM rapamycin were higher than those of the control group and the other treatment groups, while decreased maturation rates and activation rates of oocytes in 1 μM and 10 μM rapamycin groups were noted. Therefore, we selected 10 nM as the optimal rapamycin concentration for the treatment group in the subsequent experiments.

**Table 1:** Maturation rates and activation rates of mice oocytes cultured with different concentrations of rapamycin

Group	Total No. of oocytes	Maturation rate, %	Activation rate, %
Control	118	80.83 ± 1.24	53.43 ± 2.34
1 nM R	106	83.13 ± 1.76	46.57 ± 2.34
10 nM R	91	85.93 ± 0.67	62.37 ± 1.52^*^
100 nM R	103	71.93 ± 3.15	46.43 ± 2.47
1 μM R	111	60.63 ± 1.84^*^	34.33 ± 1.95^**^
10 μM R	113	66.03 ± 3.82	27.90 ± 2.36^**^

*R*: rapamycin. Asterisks indicated statistical differences compared with the control group. *P < 0.05. **P < 0.01

### IVM with 10 nM rapamycin enhanced the developmental competence of oocytes

**Fig. 1:**
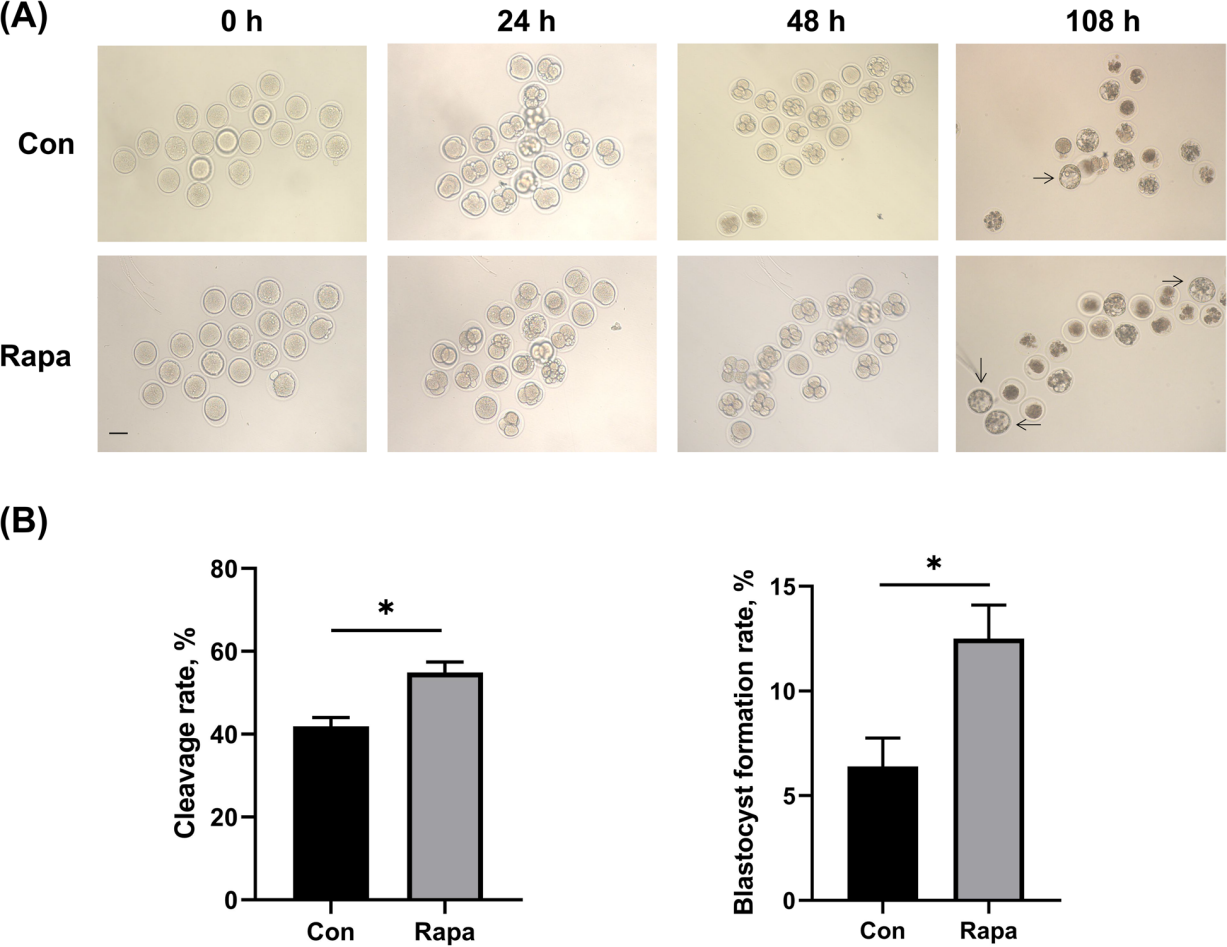
Subsequent development of PA embryos from control and 10 nM rapamycin groups. **A** The status of PA embryos in both groups at 0 h, 24 h, 48 h, and 108 h of culture. The blastocysts were indicated with black arrows. Scale bar, 50 μm. **B** The comparison of cleavage rates and blastocyst formation rates of PA embryos from both groups. All data were expressed as mean ± SEM from three independent experiments, involving totally 126 oocytes. Con: control; Rapa: rapamycin. *P < 0.05

As shown in Table 1, significantly more mature oocytes from the 10 nM rapamycin group were activated, compared with those from the control IVM group. Following activation, the PA embryos were further cultured and evaluated. As presented in Fig. 1, both the cleavage rates and blastocyst formation rates of embryos from rapamycin group were markedly increased, compared to those in control group (cleavage rate: 54.9% ± 2.6% vs. 41.9% ± 2.1%, *P* = 0.0178; blastocyst formation rate: 12.5% ± 1.6% vs. 6.4% ± 1.4%, *P* = 0.0441).

### IVM with 10 nM rapamycin significantly improved the quality of MII oocytes

**Fig. 2:**
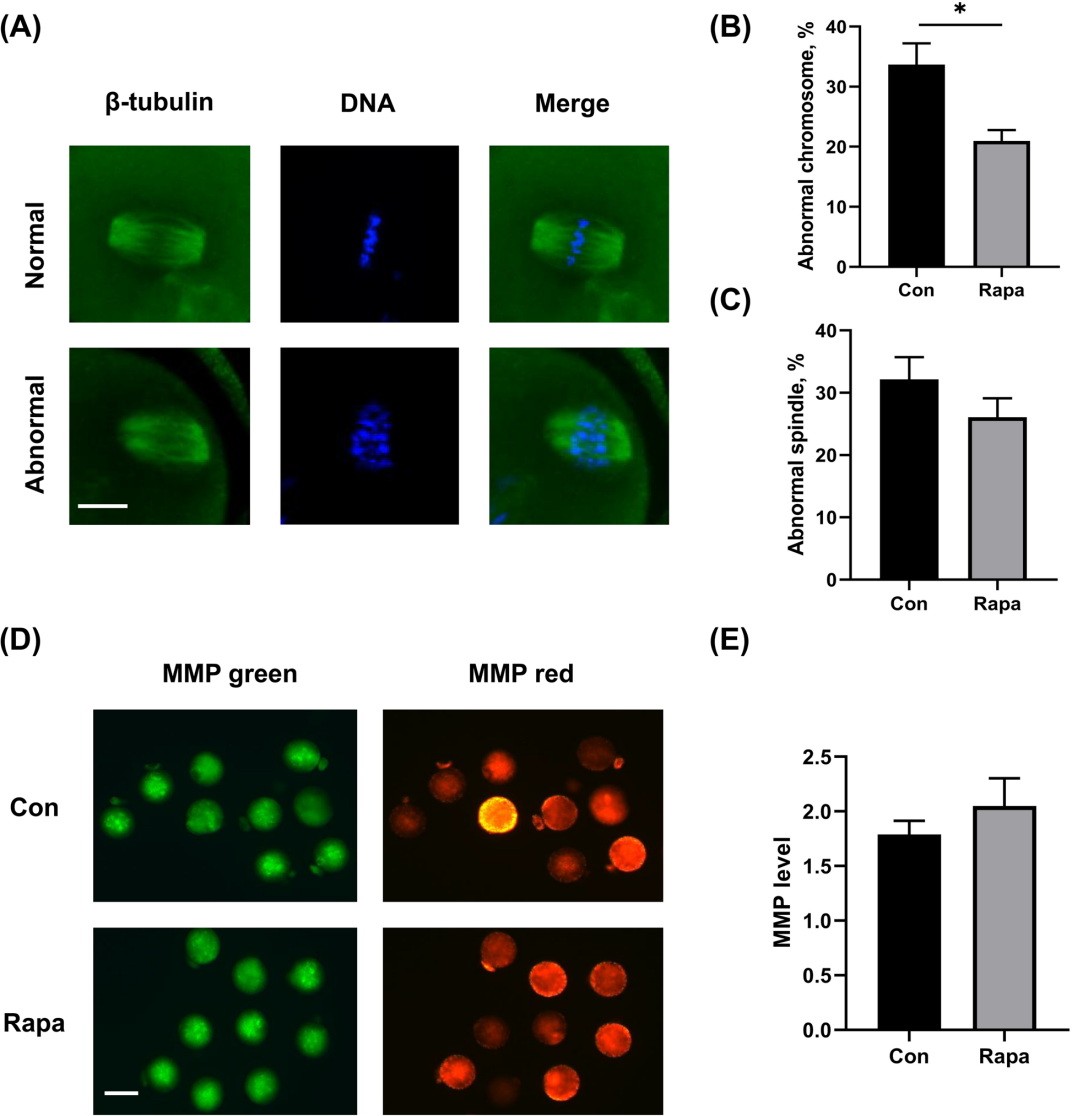
Effects of IVM with 10 nM rapamycin on the quality of MII oocytes. **A** The immunofluorescence images of normal and abnormal spindle and chromosome of IVM oocytes. Scale bar, 25 μm. **B** The percentages of oocytes with chromosome aberration. **C** The percentages of oocytes with abnormal spindle morphology. **D** The immunofluorescence images showing JC-1 staining intensity in oocytes matured in different media. Scale bar, 75 μm. **E** The relative levels of MMP (red/green fluorescence intensity) in oocytes from both groups. All data were expressed as mean ± SEM from three independent experiments, involving totally 205 oocytes. *P < 0.05

The quality of oocytes was evaluated based on spindle morphology, chromosome alignment, and mitochondrial function. As shown in Fig. 2, the percentages of MII oocytes with chromosome aberration in the 10 nM rapamycin-treated group were significantly decreased, compared with those in the control group (20.97% ± 1.81% vs. 33.70% ± 3.53%, *P* = 0.0236). As for the spindle morphology, no significant difference in the abnormal rate was observed between the two groups (26.07% ± 3.05% vs. 32.13% ± 3.58%, *P* = 0.2668). In addition, the MII oocytes were examined for levels of MMP. The results showed that the levels of MMP increased in oocytes matured with rapamycin, compared to those in control oocytes (2.05 ± 0.25 vs. 1.79 ± 0.12, *P* = 0.3942), but the difference was not statistically significant.

### IVM with 10 nM rapamycin markedly enhanced the antioxidant levels in oocytes

**Fig. 3:**
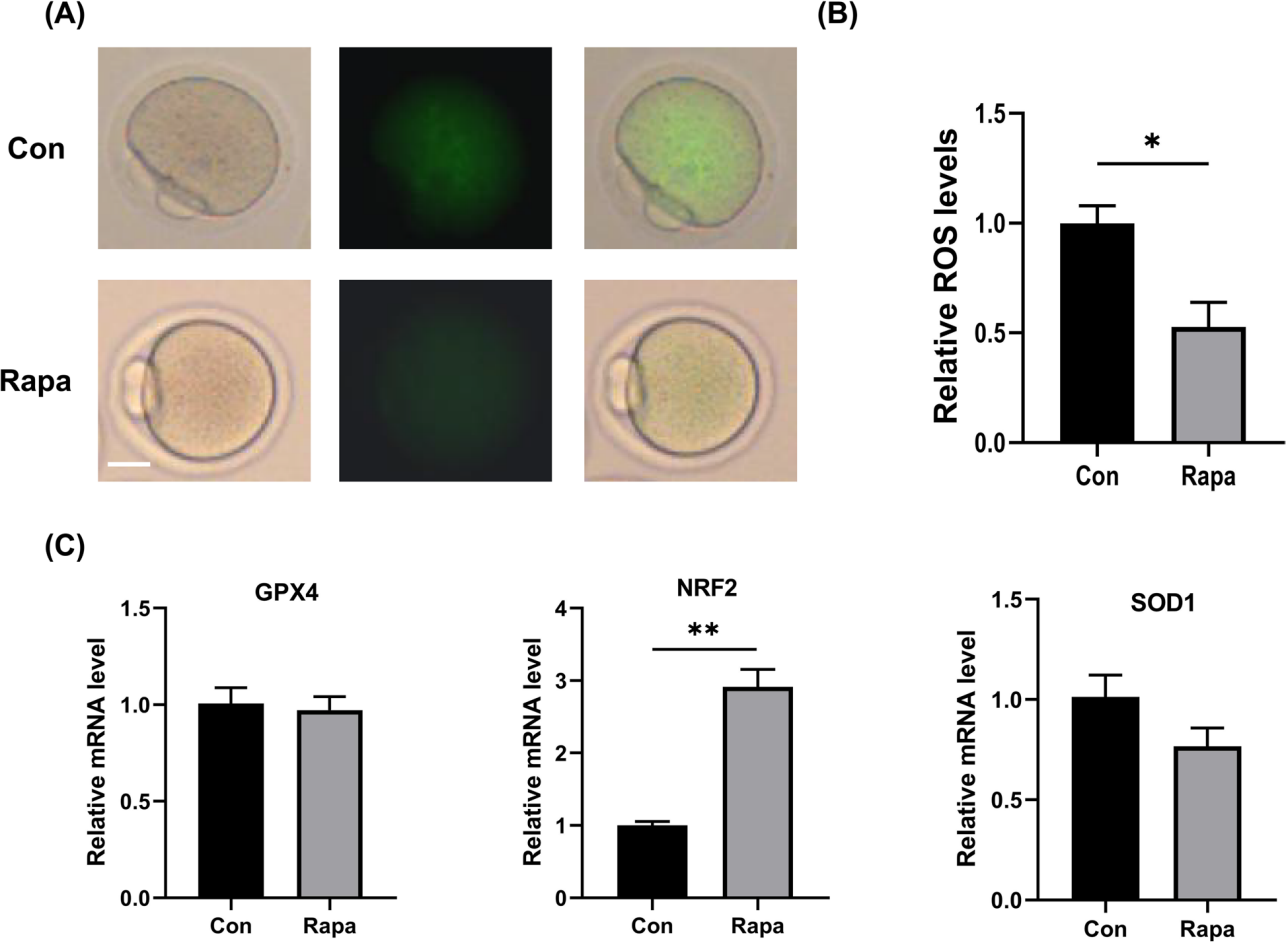
Effects of IVM with 10 nM rapamycin on the levels of ROS in IVM mice oocytes. **A** The fluorescence images of ROS. Scale bar, 25 μm. **B** The relative levels of ROS (fluorescence intensity value). **C** The mRNA levels of genes associated with anti-oxidation, including *Gpx4*, *Nrf2*, and *Sod1* in both groups. *P < 0.05, **P < 0.01

The MII oocytes were also examined for levels of ROS, and the fluorescence intensity of ROS was expressed as the fold change relative to the control. The results showed that compared to those in the control group, levels of ROS dramatically decreased in oocytes matured with 10 nM rapamycin (0.53 ± 0.11 vs. 1.00 ± 0.08, *P* = 0.0267). Furthermore, with the inhibition of rapamycin on mTORC1 pathway, the mRNA level of *Nrf2* was significantly increased in the oocytes treated with 10 nM rapamycin, while the other antioxidant genes, *Sod1* and *Gpx4*, showed no differences in expression levels between the two groups, shown in Fig. 3 and Supplemental Fig. 1.

### Rapamycin improved the DDR capacity and rescued DNA damage during IVM

**Fig. 4:**
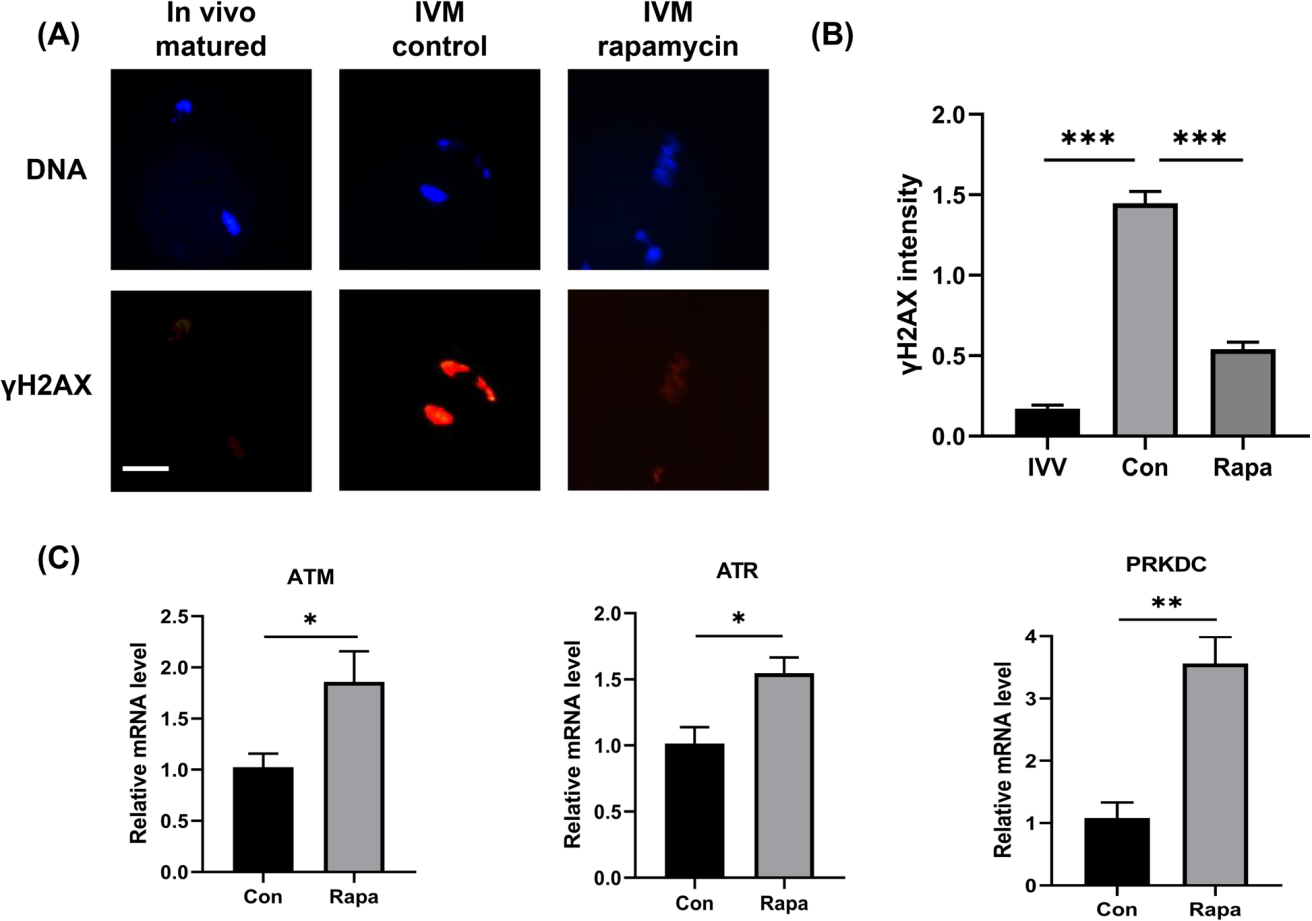
Effects of 10 nM rapamycin on the accumulation of DNA damage the expressions of genes associated with DDR during oocyte IVM. **A** The fluorescence images of DNA and γ-H2AX in oocytes matured in vivo and in vitro (with or without rapamycin). Scale bar, 20 μm. **B** The intensity of γ-H2AX normalized to the mean DNA intensity in three groups. **C** The mRNA levels of genes associated with DNA damage repair, including *Atm*, *Atr*, and *Prkdc*. IVV: in vivo; *P < 0.05; **P < 0.01; ***P < 0.001

Because the chromosome aberration in MII oocytes was significantly alleviated by 10 nM rapamycin, while the spindle assembly was not influenced, we examined γ-H2AX levels in oocytes, which in response to DSBs provides the basis for a sensitive detection for DNA damage. The γ-H2AX levels increased evidently in IVM oocytes compared with the matured ones in vivo (1.45 ± 0.07 vs. 0.17 ± 0.02, *P* < 0.0001), which was mitigated significantly by the addition of 10 nM rapamycin (0.54 ± 0.04 vs. 1.45 ± 0.07, *P* = 0.0002), as shown in Fig. 4.

Simultaneously, compared with the oocytes in the control group, the mRNA levels of DDR-associated genes *Atm*, *Atr*, and *Prkdc* increased significantly in the oocytes treated with 10 nM rapamycin. In contrast, the genes associated with spindle assembly, *Aspm*, *Numa*, and *Sirt2*, showed no significant differences between the two groups, as shown in Fig. 4 and Supplemental Fig. 1.

### mTOR activator reversed the positive effects of rapamycin on IVM

**Fig. 5:**
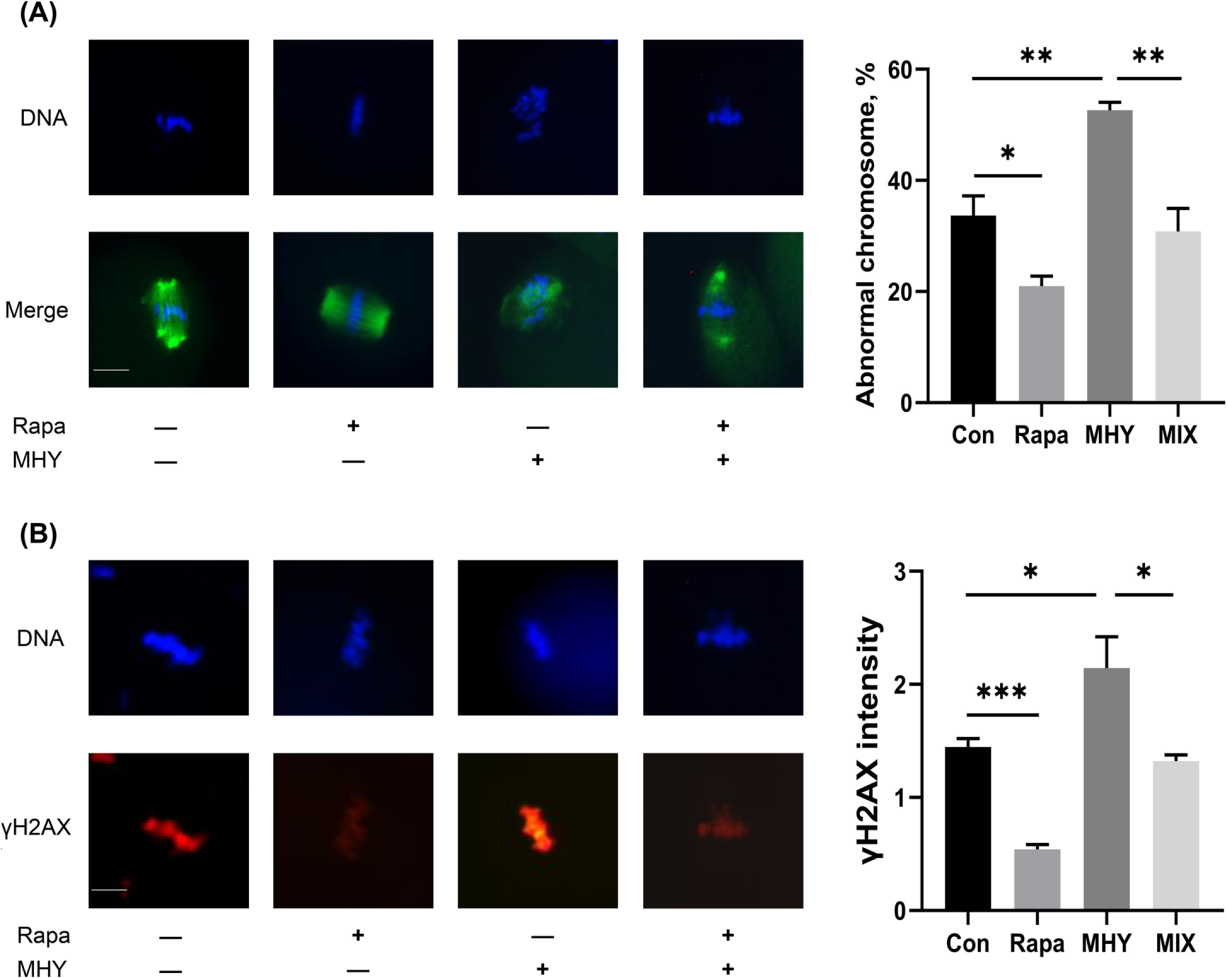
Effects of MHY1485 on chromosome alignment and DNA damage levels of IVM oocytes. **A** The immunofluorescence images of chromosome and the percentages of chromosome aberration in IVM oocytes from four groups, including control, 10 nM rapamycin-treated, 250 μM MHY1485-treated, and mix group (adding both rapamycin and MHY1485). Scale bar, 25 μm; **B** The fluorescence images of DNA and γ-H2AX in oocytes from the four groups. Scale bar, 20 μm. Con: control; Rapa: rapamycin; MHY: MHY1485. *P < 0.05; **P < 0.01; ***P < 0.001

The essential role of mTOR pathway in the improving impact of rapamycin on IVM oocytes was further investigated by adding 250 μM MHY1485, an mTOR activator. Contrary to the rapamycin-treated group, both the percentages of MII oocytes with chromosome aberration and the γ-H2AX levels in the MHY1485-treated group were significantly increased, compared with those in the control group (chromosome aberration rate: 52.63% ± 1.45% vs. 33.70% ± 3.53%, *P* = 0.0077; γ-H2AX levels: 2.15 ± 0.28 vs. 0.54 ± 0.04, *P* = 0.0370). Simultaneously, combination of rapamycin and MHY1485 significantly rescued the negative effects of MHY1485 on the chromosome alignment and DNA damage levels (chromosome aberration rate: 30.83% ± 4.17% vs. 52.63% ± 1.45%, *P* = 0.0078; γ-H2AX levels: 1.32 ± 0.05 vs. 2.15 ± 0.28, *P* = 0.0433), as shown in Fig. 5.

## Discussion

In the current study, IVM of mice oocytes was performed with or without the addition of rapamycin, following which the developmental competence and quality of matured oocytes were evaluated. The results indicated that 10 nM rapamycin treatment could increase the maturation rate, activation rate of IVM oocytes, cleavage rate, and blastocyst formation rate of the PA embryos, and simultaneously improve the chromosome arrangement and reduce the ROS level of matured oocytes. Mechanistically, these improvements may be related to the effect of rapamycin on the DDR capacity of IVM oocytes.

The effects of rapamycin on the developmental competences of IVM oocytes have been investigated in previous studies, although with different results. Yang et al. added rapamycin at concentrations of 0.5-10 μg/μl (i.e. 0.55-10.94 mM) into the IVM system of mice oocytes and found that the GVBD rate and PB1 extrusion rate were significantly reduced when the concentration reached or exceeded 3.3 μg/μl [13]. Later studies, however, showed opposite conclusions after reducing the rapamycin concentrations. Several studies demonstrated that 1 nM rapamycin could increase the maturation rate and improve the subsequent developmental potential of IVM porcine oocytes, especially the poor-quality oocytes [16–18]. In the experiments using bovine oocytes, the results were varied. Kordowitzki et al. added 0.1 nM, 1 nM, 10 nM, and 100 nM rapamycin to IVM medium, and found that the maturation rate was the highest in the 1 nM group and the lowest in the 100 nM group, but the maturation rate, cleavage rate, and blastocyst formation rate of IVM oocytes were not significantly different between the 1 nM rapamycin group and control group [19]. In another study, bovine COCs were divided into high-quality and low-quality groups according to morphological evaluation. The results showed that the addition of 100 nM rapamycin in IVM medium significantly increased the cleavage rate of low-quality bovine COCs after maturation and fertilization, but not the blastocyst formation rate [20].

Taken together, the effect of rapamycin on oocyte IVM outcomes seemed to be largely determined by its concentration. And our results provided a detailed exhibition of the dose-dependent effect of rapamycin on IVM oocytes, that high concentrations of rapamycin inhibited oocyte maturation, while lower concentrations played a promoting role, with 10 nM showing the optimal effect in mice oocytes, which were generally consistent with previous reports. Alterations in the effect may be attributed to the diversity of cellular activities implicated in the mTOR pathway and the presence of negative feedback mechanisms [21–23].

We selected 10 nM as the optimal concentration of rapamycin to continue the later experiments in our study. Following IVM with or without 10 nM rapamycin, the matured oocytes were collected for PA and subsequent developments of the PA embryos were assessed. Although all the matured oocytes completed the first meiosis morphologically, those from the control group presented rather poor developmental potential, which was dramatically improved with the addition of 10 nM rapamycin. It seemed that 10 nM rapamycin not only increased the proportion of mature oocytes but may also positively affect their qualities, which was elucidated by our latter experiments.

The most significant effect of rapamycin on the quality of IVM oocytes was the improvement of chromosome alignment. Both abnormal spindle assembly and DNA damage may cause chromatin disarrangement or even fragmentation, which leads to oocyte developmental disorders [24, 25]. Given that there was no significant difference in spindle morphology between the two groups of oocytes, we reasoned that the improved chromosome alignment by rapamycin might be attributed to its effect on DDR. Considering the γH2AX is a sensitive marker for the detection of DNA damage [26], we examined γH2AX levels in oocytes before and after IVM. While very little fluorescence signal of γH2AX was observed in in vivo matured oocytes, IVM process evidently increased γH2AX levels, which were significantly reduced by addition of 10 nM rapamycin. Noteworthily, these impacts were reversed to some extent after the mTOR activator was applied, indicating that rapamycin exerted the positive effects by inhibiting mTOR.

The protective effect of rapamycin against DNA damage was further confirmed by the enhancement of the DDR-associated genes expression. Ataxia telangiectasia mutated (ATM), DNA protein kinase (DNA-PK, encoded by *Prkdc*), and ataxia telangiectasia and Rad3 related (ATR), are three proteins from the PIKK family, which are recruited to the site of DNA damage and subsequently phosphorylate H2AX, activate cell checkpoints, and coordinate DNA repair through homologous recombination (HR) or non-homologous end joining (NHEJ) [27–29]. In recent years, increasing evidence showed that the mTORC1 pathway was involved in the DNA damage response [30]. Shen et al. proved in the context of childhood sarcoma that mTOR signaling suppressed *Atm* mRNA via the S6K pathway, and inhibition of mTOR enhanced the expression of *Atm* [31]. Also, exposure of cancer cells to rapamycin can increase DNA-PK activity and promote NHEJ [32]. In agreement with previous studies regarding cancer cells, our study demonstrated that the inhibition of mTOR by 10 nM rapamycin during oocyte IVM significantly increased the expressions of these DDR-associated genes (*Atm*, *Atr*, and *Prkdc*), improved the DNA repair capacity of the oocytes, and thus prevented the DNA damage accumulation.

Previous studies have demonstrated different fates of oocytes with DNA DSBs in vitro and in vivo. While oocytes with severe DNA DSBs undergo apoptosis in vivo, those in vitro still could undergo GVBD and complete meiosis I reaching the MII stage, which might be partially explained by the reduced ability to activate ATM and affect the cell cycle checkpoints [33, 34]. Our results proved the promoting effect of rapamycin on the DDR process in IVM oocytes, providing a promising method to improve the quality of IVM oocytes and ultimately ameliorate the reproductive outcomes of patients undergoing IVM.

Another benefit of rapamycin treatment during oocytes IVM was its anti-oxidant effect. Accompanied with the down-regulation of mTORC1 pathway, significantly decreased ROS levels in IVM oocytes treated with 10 nM rapamycin were observed. Simultaneously, the expression of the nuclear transcription factor E2-related factor 2 (Nrf2), a master transcription factor of many antioxidant genes and phase II detoxifying enzymes [35], was increased in these oocytes. The relationship between mTOR and NRF2 pathway remained unclear, with little literature reporting inconsistent conclusions in different cell types. Shibata et al. reported that oncogenic Nrf2 mutation induces dependence on the mTOR pathway during carcinogenesis [36]; Sun et al. demonstrated that Nrf2 regulates mTOR during histone deacetylase inhibitor (HDACi)-induced autophagy and inhibition of this pathway could enhance HDACi-mediated cell death [37]. Conversely, Wang et al. found that in mouse skin fibroblasts, rapamycin increased the levels of Nrf2 in a dose-dependent manner and lowered the levels of Keap1 [38], a protein that triggers Nrf2 degradation [39]. Furthermore, Qiao et al. claimed that mTOR was located upstream of Nrf2 in glomerular mesangial cells, and bergenin enhanced Nrf2 activity by inhibiting mTOR pathway [40]. Our study proved for the first time that the cross-linking effect of these two pathways also existed in oocytes matured in vitro, and rapamycin might reduce the ROS level by inhibiting mTOR and activating Nrf2 during IVM. The complex interactions between mTOR and Nrf2 pathway require further exploration.

## Conclusion

In conclusion, our study revealed the dose-dependent effect of rapamycin during oocyte IVM, confirmed the antioxidant function of rapamycin, and initially presented the effects of rapamycin on DNA damage level and DDR capacity of the IVM oocytes. Based on the important role of DNA damage repair in the development of IVM oocytes, the application of rapamycin in IVM would be a promising method for improving IVM outcomes. In the future, more human oocyte-based studies need to be carried out subsequently and the effect and safety of rapamycin should be further validated to advance its clinical application in the field of IVM.

## Supplementary Information

**Additional file 1: Supplemental Figure 1.** Effects of 10 nM rapamycin on the expressions of genes in mTORC1 pathway and genes associated with spindle assembly in IVM oocytes. (A)-(C): The mRNA levels of genes on the mTORC1 pathway, including *Mtor*, *S6k1*, and *4ebp1.* (D)-(F): The mRNA levels of genes associated with spindle assembly, including *Aspm*, *Numa*, and *Sirt2*. Con: control; Rapa: rapamycin. *P < 0.05; **P < 0.01.

**Additional file 2: Supplemental Table 1.** Primers used for real-time PCR.

## Data Availability

All data generated or analysed during this study are included in this published article.

## Abbreviations

IVM: In vitro maturation
IVF-ET: In vitro fertilization-embryo transfer
PCOS: Polycystic ovary syndrome
OHSS: Ovarian hyperstimulation syndrome
GVBD: Germinal vesicle breakdown
PB1: First polar body
COH: Controlled ovarian hyperstimulation
DSBs: Double-strand breaks
DDR: DNA damage repair
mTOR: Mammalian target of rapamycin
ROS: Reactive oxygen species
MMP: Mitochondrial membrane potential
COC: Cumulus-oocyte complex
ATM: Ataxia telangiectasia mutated
ATR: Ataxia telangiectasia and Rad3 related
DNA-PK: DNA protein kinase
HR: Homologous recombination
NHEJ: Non-homologous end joining
