# Supplementary figures and images for: Rapamycin improves the quality and developmental competence of mice oocytes by promoting DNA damage repair during in vitro maturation
Source: Reprod Biol Endocrinol. 2022 Apr 18;20:67. doi: 10.1186/s12958-022-00943-0 (PMC9014618; doi:10.1186/s12958-022-00943-0)

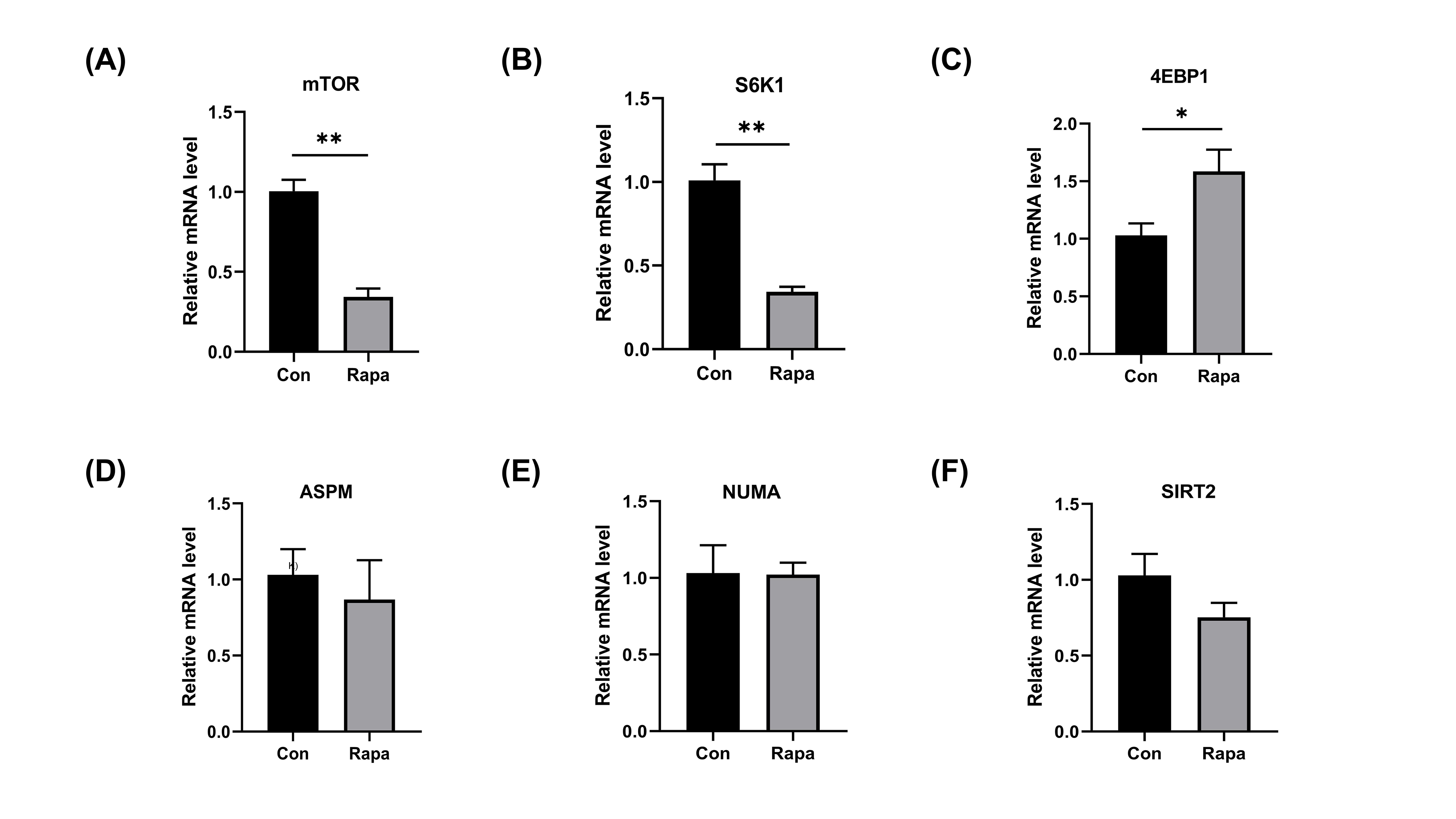

Supplement: Supplementary file 1 — Additional file 1: Supplemental Figure 1. Effects of 10 nM rapamycin on the expressions of genes in mTORC1 pathway and genes associated with spindle assembly in IVM oocytes. (A)-(C): The mRNA levels of genes on the mTORC1 pathway, including Mtor, S6k1, and 4ebp1. (D)-(F): The mRNA levels of genes associated with spindle assembly, including Aspm, Numa, and Sirt2. Con: control; Rapa: rapamycin. *P < 0.05; **P < 0.01. [file 12958_2022_943_MOESM1_ESM.tif]
