# Supplementary material for: Rapamycin improves the quality and developmental competence of mice oocytes by promoting DNA damage repair during in vitro maturation
Source: Reprod Biol Endocrinol. 2022 Apr 18;20:67. doi: 10.1186/s12958-022-00943-0 (PMC9014618; doi:10.1186/s12958-022-00943-0)
Supplement: Supplementary file 2 — Additional file 2: Supplemental Table 1. Primers used for real-time PCR. [file 12958_2022_943_MOESM2_ESM.docx]

**Supplemental Table 1 Primers used for real-time PCR**

| Gene | GenBank accession no. | Primer sequence | Annealing temp. |
| --- | --- | --- | --- |
| MTOR | NM_020009 | F: CACCAGAATTGGCAGATTTGC | 60℃ |
|  |  | R: CTTGGACGCCATTTCCATGAC |  |
| S6K1 | NM_001114334 | F: AGACACAGCGTGCTTTTACTT | 60℃ |
|  |  | R: GTGTGCGTGACTGTTCCATCA |  |
| 4EBP1 | NM_007918 | F: GGGGACTACAGCACCACTC | 50℃ |
|  |  | R: GTTCCGACACTCCATCAGAAAT |  |
| ASPM | NM_009791 | F: CTATCCGACGAAGCCTTAGAG | 55℃ |
|  |  | R: AGGTGAGCAGGTGATGAAGC |  |
| SIRT2 | NM_001122766 | F: CCAAGAAGGCTTACAGGG | 60℃ |
|  |  | R: CAGGGGAGATGGTAGTGC |  |
| NUMA | NM_006185 | F: CACTCAATGCCACCATCCAG | 50℃ |
|  |  | R: TGCTTCTCCGCTACCTCCTT |  |
| GPX4 | NM_001367995 | F: AGGAGCCAGGAAGTAATC | 50℃ |
|  |  | R: TCAATGAGAAACTTGGTAAA |  |
| NRF2 | NM_010902 | F: CAGTGCTCCTATGCGTGAA | 60℃ |
|  |  | R: AGCGGCTTGAATGTTTGT |  |
| SOD1 | NM_011434 | F: GCTGTACCAGTGCAGGTCCTCA | 60℃ |
|  |  | R: CATTTCCACCTTTGCCCAAGTC |  |
| ATM | NM_007499 | F: TGCTACCAAGGTCTACGA | 50℃ |
|  |  | R: GCAACTCCACCACAATCT |  |
| ATR | NM_019864 | F: GAATGGGTGAACAATACTGCTGG | 60℃ |
|  |  | R: TTTGGTAGCATACACTGGCGA |  |
| DNAPK | NM_011159 | F: GCCTTTTCGTCTAACCCG | 50℃ |
|  |  | R: ATCCTCCTTTTCTCAGCATT |  |
| GAPDH | NM_001289726 | F: ATTCAACGGCACAGTCAA | 60℃ |
|  |  | R: TTAGTGGGGTCTCGCTCC |  |
